# Fungal Biodeterioration, Aflatoxin Contamination, and Nutrient Value of “Suya Spices”

**DOI:** 10.1155/2016/4602036

**Published:** 2016-03-22

**Authors:** Segun Gbolagade Jonathan, Mary Adejoke Adeniyi, Michael Dare Asemoloye

**Affiliations:** Food & Environmental Mycology/Biotechnology Unit, Department of Botany, University of Ibadan, Ibadan 200284, Oyo State, Nigeria

## Abstract

This work aimed to analyze the nutrient values, examine the biodeteriorating fungi biota, and analyze the mycotoxin contents of “Suya spices.” Fungi with highest percentage occurrence on all the samples are* Aspergillus niger*,* Aspergillus flavus*,* Aspergillus parasiticus*,* Aspergillus ochraceus*,* Fusarium *sp.,* Rhizopus stolonifer*, yeast, and* Trichoderma koningii*. Nutrient composition of the samples is significantly different statistically (*P* < 0.05) with high protein (9.53% to 13.17%), fiber (9.27 to 13.17%), carbohydrate (46.27% to 50.90%), and ash (8.47% to 9.70%) contents but low moisture (9.03% to 9.47%) and fat (9.77% to 13.53%) contents. Aflatoxin analysis of the samples revealed that they all contain aflatoxin in varying amount but no detectible aflatoxin content in the control. 59.54% of the detected aflatoxin is aflatoxin B_1_ with highest recorded in Agbowo, Mokola, and Sango samples (i.e., 28.03, 22.44, and 13.8 *μ*g/kg, resp.). 4.78% of the aflatoxin is aflatoxin B_2_ which is only found in Sango and Mokola samples (3.59 and 2.6 *μ*g/kg, resp.). 32.76% of aflatoxin is aflatoxin G_1_ with the highest found in Agbowo and Mokola samples (i.e., 18.63 and 10.41 *μ*g/kg, resp.). 2.93% of the aflatoxin is aflatoxin G_2_ which is only detected in Sango and Agbowo samples (i.e., 1.19 and 2.65 *μ*g/kg, resp.).

## 1. Introduction

“Suya spices” are a Nigerian indigenous spice commonly used on roasted meat (barbecued meat) to give it a unique desired taste and it originated from the Hausa speaking people in the country. It is the special blend of peppers and spices that is used to make Nigerian Suya (Nigerian shish kebab, roasted skewered meat with a particularly African twist). The spice consists of grinded pepper (*Capsicum* sp.),* Xylopia aethiopica*,* Piper guineense*, and* Monodora myristica* [1]. These spices help in adding aroma and flavor to the barbecued meat (Suya). Though Suya is prepared by the Hausas, its consumption transcends the borders of ethnicity, especially among the elites during relaxation period. Early studies showed that spices used in the preparation of Suya may contain high population of bacteria and fungi, which remain viable even at the time of marketing [2, 3], also according to [4]. If the spice is not hygienic or not properly kept, it may be contaminated by microorganism and may cause health hazard conditions like food poisoning. Large groups of microorganism have been isolated from some spices and some vended foods of which fungal groups are notable. Despite the fact that some spices and herbs have been documented to have antimicrobial activities, the quantity of added spices to food may not be enough to adequately inhibit microbial contaminations most especially fungi; even the antimicrobial activities of these spices may vary widely depending on the type of spices [5].

Microbial contamination has been reported to be the cause of food illnesses and spoilage [6–8] and many of these microbes are fungi. Fungi are ubiquitous or cosmopolitan (i.e., they can be present everywhere, in the air, water, and soil, and even on man and inside him) as explained by Jonathan et al. [8]. They are group of organisms known to be good “biodegraders” of waste, many of which have different characteristic mode of converting waste of living and dead tissues of various products such as plants products, agricultural products, wood and paper products, dead animal tissues, and chemical waste [9, 10]. Most fungi are generally more tolerant to high concentrations of many pollutants than bacteria; this explains why fungi have been investigated more extensively for their biodegradation and bioremediation capacities dated back to the mid-1980s [8]. Many groups of fungi mostly filamentous (Deuteromycota) fungi such as* Penicillium*,* Aspergillus, Fusarium*,* Rhizopus*, and* Mucor* have been successfully isolated from spices and some street vended foods [2, 5, 6, 10–13]. These fungi are involved in food spoilage, an activity which is common among the molds which results in the reduction of food value/nutrient of the particular food. Some of these fungi may introduce metabolites into these foods under favourable environmental condition, in order to prevent other organisms including humans from eating those [7], hence making the food to be poisonous.

The metabolites are called mycotoxin which literally means “fungal poison thereby referred to as mycotoxin.” Aflatoxins are the most deadly mycotoxins, they are produced by* Aspergillus* species and are known to be one of the most deadly carcinogens due to detrimental effects they can exert on their consumers, and this is also confirmed by the International Agency for Research on Cancer (IARC) [14]; they explained further that there is sufficient evidence in humans for the carcinogenicity of natural occurring aflatoxins and classified them as Group 1 carcinogens. There are five different types of aflatoxins that exist in nature; they are aflatoxin B_1_ (AFB1), aflatoxin B_2_ (AFB2)_,_ aflatoxin G_1_ (AFG1)_,_ aflatoxin G_2_ (AFG2), and aflatoxin M_1_ (AFM1), respectively. Aflatoxins are toxins produced mainly by two* Aspergillus *species, that is,* A. flavus* and* A. parasiticus*, and the categories of foods they contaminate are cereals and cereals' products; herbs and spices; nuts and oil seeds; meat and poultry products; animal feeds; and milk and milk products. “Suya spices” fall into the group of foods targeted by aflatoxins.

In Nigeria, mycotoxin contamination of cereals (rice), grains (maize), and seeds (cocoa) has raised a lot of concern for food safety [12] as these foods, especially rice and maize, are not only eaten directly, but also used in the production of various forms of indigenous foods like* ogi*,* eko*,* tuwo*,* kunu*,* donkwa*, and* masa*, among others. Bankole et al. [15] noted that the mycotoxin called aflatoxin has received most of the attention in food products in West African subregion, while there are few researches carried out on other mycotoxins such as fumonisin and ochratoxin A. However, there have been some recent studies on the mycotoxins present in food products from Nigeria, especially maize, rice, groundnuts, guinea corn, sorghum, cocoa, and cocoa-based beverages [6–8, 10, 13, 16–20].

The main objectives of this study were to isolate the fungi associated with biodeterioration of samples of “Suya spices”; check whether the food value has been spoilt or affected due to the activities of fungal organisms; ascertain and quantify the presence of aflatoxins (fungi metabolites) in the samples of “Suya spices” collected from five different locations in Ibadan, Oyo state of Nigeria, and compare them with aseptically prepared ones from the laboratory and to establish (if detected) how these aflatoxins pose threat to human.

## 2. Materials and Methods

### 2.1. Sources of Materials for “Suya Spice” Production

Pepper (*Capsicum* sp.),* Piper guineense,* Maggi, and salt were purchased from Bodija market, Ibadan, Nigeria.

### 2.2. Collection of “Suya Spice” Samples

Five samples of “Suya spices” (500 g each) were purchased from different locations in Nigeria. These locations are known to be famous in Nigeria, well-populated, and active in marketing activities. These locations are Agbowo, Sabo, Mokola, Ojoo, and Sango. Also a laboratory sample was used as control for each of “Suya spices.”

### 2.3. Preparation of Laboratory Sample

Dry pepper (*Capsicum* sp.) and* Piper guineense* were ground to fine powder and were mixed with Maggi and salt.

### 2.4. Isolation of Fungi Biota

The samples were brought to the department and each sample was homogenized and 1 g was dissolved in 10 mil distilled water and then 1 mil of the mixture was added to 9 mil of sterilized distilled water followed with serial dilution at 10^−1^ to 10^−6^, 1 mil of dilution 10^−6^ inoculated into the medium using direct inoculation method. The medium used for the isolation was potato dextrose agar (PDA) in plate and incubated at 30 ± 2°C for 5 to 7 days according to the procedure described by Jonathan and Olowolafe [21]. The cultures were examined under microscope for fruiting bodies and hyphae to determine the presence of fungi.

### 2.5. Characterization and Identification

Characterization and identification of isolated fungi were done based on the morphological characters and microscopic structures of the fungi [6, 7, 13]. The colonies of the organism were observed for peculiar characteristic colonial morphology and this was done using the following listed features:

- Colonial appearance.
- Rate of growth followed at regular intervals.
- Texture of colonies.
- Colour of colonies.
- Reverse side or colour of underside.

Microscopic morphology and type of asexual spores produced were studied through use of photomicrograph and identified by reference to the compendium of soil fungi [22].

### 2.6. Proximate Analysis

Samples of Aadun were taken for proximate analysis and the determination of various parameters was carried out at KAPPA laboratories, Ibadan. The moisture, crude protein, crude fat, crude fiber, and total ash were determined using AOAC [23] methods while the carbohydrate was determined by difference.

### 2.7. Aflatoxin Analysis

The aflatoxin analysis was carried out in pathology laboratory of International Institute of Tropical Agriculture (IITA), Ibadan, using the HPLC methods as described by Oluwafemi and Ibeh [24]. The HPLC is made up of LDC, with Milton Roy, Constametric 1 pump, and a Lichrosorb RP-18 column (Merck Hibar) with particle size of 5 *μ*m, length of 125 mm, and inside diameter of 4 mm. The pump pressure is 60 MPa and the injector was of an automatic type (Rheotype Gilson Abimed Model 231). The detector had a fluorescence spectrophotometer (Shimadzu RF 535, gamma excitation 365 mm, and gamma emission 444 nm) and the flow rate was 1 mL per minute and the injection volume was 50 *μ*L with the use of mobile phase containing water/acetonitrile (75 : 25) with flow rate 1.2 mL min^−1^ for 20 min.

50 gram of each sample of “Suya spice” was defatted by extraction with N-hexene Soxhlet-type extractor and the defatted residue was extracted with ethyl acetate (three times, 50 mL/each). The extracts were combined, dried over anhydrous sodium sulphate, filtered and then concentrated under vacuum to near dryness, transferred into brown glass vial, and evaporated under nitrogen stream. For cleaning up the crude extracts, the crude extract was suspended in 1 mL chloroform and applied to 14 × 0.8 cm column containing 2.5 Kiesel gel 60 and 70/230 silica gel. The aflatoxin analysis was done using Lichrosorb RP-18 column. The quantitative determination of the aflatoxins was carried out compared with standard aflatoxin B_1_ (Sigma).

## 3. Result and Discussion

**Figure 1:**
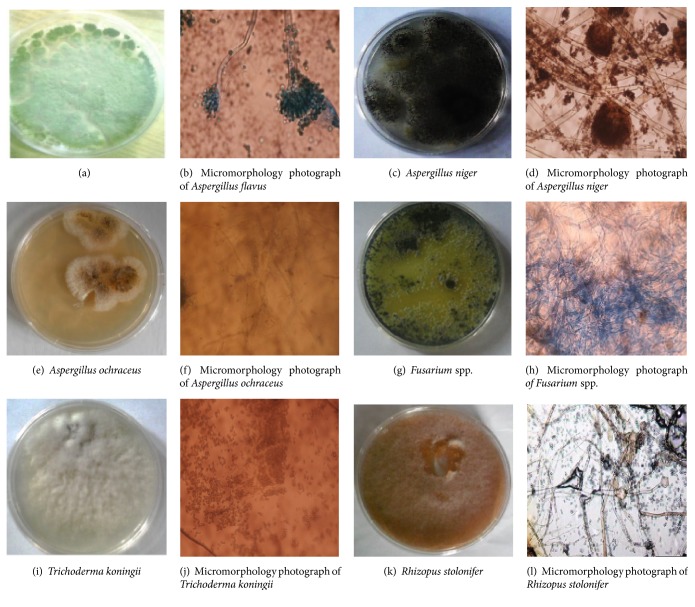


The fungal isolates from samples of “Suya spices” are mostly of filamentous fungi most of which belong to the species* Aspergillus*. The fungi that appear mostly on the vended samples (i.e., are isolated from all the market samples) are* Aspergillus flavus*,* Aspergillus niger*,* Aspergillus ochraceus*,* Aspergillus parasiticus*,* Fusarium* sp.,* Rhizopus stolonifer*, yeast, and* Trichoderma koningii* (Figures 1(a)–1(l) with their microscopic views) and this is in agreement with the findings of Fabian et al. [2]; Yasair and Williams [3]; Giese [5]; Nwaiwu and Imo [11]; Kumar et al. [12]; Jonathan et al. [6, 7, 13]; and olayiwola et al. [10] who isolated similar organisms from spices and some other street vended foods.

Mokola and Agbowo samples were found to be highly contaminated with fungal organisms. These fungi were found to be responsible for the depreciation of the food value of the samples collected as explained by Table 1, although the colour and aroma were unaffected for a period of time (over one month); that is, no sign of deterioration was noticed on the outside.

The aflatoxin analysis carried out on the samples revealed that all the samples contain varying amount of aflatoxins except the control, 33% of the samples contain aflatoxin B_2_, 67% of the samples have aflatoxin G_1_ while 33% of the samples contain aflatoxin G_2_, and the control was found to be free of aflatoxins (the four types; B_1_, B_2_, G_1_, and the G_2_).

**Figure 2:**
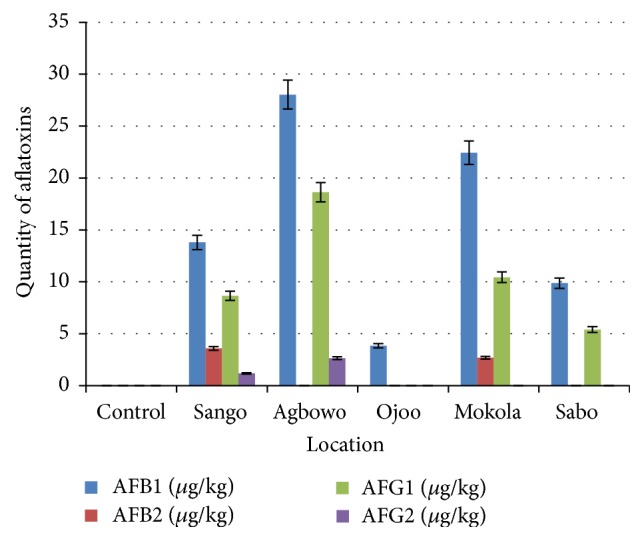
Aflatoxin Quantification of samples of “Suya spices” with percentage bars.

Aflatoxin B_1_ was found in all the samples except the control (Figure 2), but at various concentrations; this can be attributed to the fact that when aflatoxin is produced by either* A*.* flavus* or* A*.* parasiticus*, aflatoxin B_1_ is the first metabolite released before others (aflatoxins B_2_, G_1_, and G_2_) depending on the production rate [25]. To arrange samples based on the concentration of aflatoxin they contain (from the highest to the lowest), we have Agbowo; Mokola; Sango; and Sabo, respectively. Of all the samples, Agbowo and Mokola samples were found to contain lethal dosage as they contain dosage of aflatoxin B_1_ which is beyond tolerable limit (the standard being 20 *μ*g/kg for human consumption).

Aflatoxin B_2_ was detected in only samples from Sango and Mokola but is below or within the confines of tolerable limit while aflatoxin G_1_ is found in all the samples like aflatoxin B_1_ but the concentrations in the individual sample are below the limit except the amount contained in Agbowo sample (18.63 *μ*g/kg) which can be considered as close to the danger limit. Aflatoxin G_2_ was found in Sango and Agbowo samples where the concentration is very low.

Table 2 shows the effect of the aflatoxin concentration on each of the food products, Aadun (cereal based product) and “Suya spices” (pepper based product), and this indicates that the aflatoxins have significant effect on the food products. The aflatoxin analysis of the samples revealed that they all contain aflatoxins in varying amount and there were no detectible aflatoxin contents found in the control. 59.54% of the aflatoxins are aflatoxin B_1_ with highest recorded in Agbowo, Mokola, and Sango samples (i.e., 28.03, 22.44, and 13.8 *μ*g/kg, resp.). 4.78% of the aflatoxins are aflatoxin B_2_ which are only found in Sango and Mokola samples (3.59 and 2.6 *μ*g/kg, resp.). 32.76% of aflatoxins are aflatoxin G_1_ with the highest found in Agbowo and Mokola samples (i.e., 18.63 and 10.41 *μ*g/kg, resp.). 2.93% of aflatoxins are aflatoxin G_2_ which was detected only in Sango and Agbowo samples (i.e., 1.19 and 2.65 *μ*g/kg, resp.).

From Figure 2 and Table 2, it is observed that vended “Suya spice” collected from Agbowo had the highest number of detected aflatoxins followed by Mokola and Sango samples while Ojoo and Sabo had the least aside control that had no detectable aflatoxin content.

Results of this conform a statement credited to APS [26] that the main factor responsible for mycotoxin production is the interaction between the fungi, its host, and the environmental condition as the concentrations of the aflatoxin vary with the location. The appropriate combination of these conditions determines the invasion and colonization of the substrate and the type and quantity of aflatoxin produced. However, a suitable substrate is required for optimum fungal growth and subsequent toxin production, although the precise factor(s) that initiates toxin formation is not well understood but a proper transport, handling, and storage of prepared food are often critical to the safety of street vended foods.

## 4. Conclusion

The results from this study can be linked to some factors as follows:

- The air flora of the location considering the possibility of spores being carried by dust as the two areas are usually busy especially the Agbowo and Mokola sample that was collected when the construction of the flyover bridge at Mokola was still on.
- Low sanitary precautions taken by the food handlers.
- The raw materials used in making the “Suya spices” which are majorly pepper which have not been well screened by plant breeders against aflatoxins (Aflasave).

Therefore, we recommend safe handling of the spice and proper hygienic measures especially in Agbowo and Mokola areas as aflatoxin concentrations above tolerant limit were detected in these locations. We also recommend proper storage system of the vended spice as the mold spores are abundant in the air. Continuous check for aflatoxin detection on food materials should be encouraged as this may serve as checkmating of the safety of consumed food.

## Figures and Tables

**Table 1:** Proximate analysis of “Suya spices.”

Location	Moisture content (%)	Protein content (%)	Fat content (%)	Ash content (%)	Fiber content (%)	Carbohydrate content (%)
Control	9.10^c^	9.53^e^	11.17^c^	8.77^c^	13.17^a^	48.30^b^
Agbowo	9.73^a^	10.80^d^	12.47^b^	9.70^a^	9.77^e^	47.53^c^
Sabo	9.03^c^	9.63^e^	13.53^a^	7.93^e^	9.27^f^	50.60^a^
Mokola	9.43^b^	13.17^a^	10.53^d^	9.37^b^	11.30^c^	46.27^d^
Ojoo	9.47^b^	12.33^b^	10.17^e^	9.30^b^	12.43^b^	46.30^d^
Sango	9.17^c^	11.20^c^	9.77^f^	8.47^d^	10.50^d^	50.90^a^

Each value is a mean of three replicates. Values in the same column with different letters as superscripts are significantly different by Duncan multiple range test (*P* ≤ 0.05).

**(a):** 

Location	Aflatoxin concentration (*µ*g/kg)				
	AFB1	AFB2	AFG1	AFG2	Total
Control	0	0	0	0	0
Sango	13.8	3.59	8.66	1.19	27.24
Agbowo	28.03	0	18.63	2.65	49.31
Ojoo	3.85	0	0	0	3.85
Mokola	22.44	2.69	10.45	0	35.58
Sabo	9.88	0	5.41	0	15.29
Total	78	6.28	43.15	3.84	131.27

% aflatoxin	59.54	4.78	32.76	2.93	100.01

**(b):** 

	AFB1		AFB2		AFG1		AFG2	
	MS	SE	MS	SE	MS	SE	MS	SE
Suya spices	230.65^*∗∗*^	1.31	5.40^*∗∗*^	0.34	100.03^*∗∗*^	0.25	2.39^*∗∗*^	0.12

*∗* means *P* < 0.05, significant, and *∗∗* means *P* < 0.01, highly significant.
